# Dysregulation of Plasma Nonessential Amino Acids in Early‐Stage Type 2 Diabetes Mellitus

**DOI:** 10.1155/jnme/2912642

**Published:** 2026-08-17

**Authors:** Abdullah Abbas Hamzah Al-Rubaye, Walaa Esmail Jasim, Ahmed A. Mohsin

**Affiliations:** ^1^ Department of Medical Laboratory Technology, College of Health and Medical Technology, Southern Technical University, Basra, Iraq, stu.edu.iq; ^2^ Department of Medical Laboratory Technology, College of Health and Medical Technology, Middle Technical University, Baghdad, Iraq, mtu.edu.iq

**Keywords:** biomarkers, diagnosis, hyperglycemia, insulin resistance, management, obesity, prediabetes

## Abstract

**Background:**

Type 2 diabetes mellitus (T2DM) is a chronic metabolic disorder characterized by hyperglycemia due to insufficient insulin production or the body’s poor response to insulin (insulin resistance). Amino acids are closely associated with glucose dysregulation, and disturbances of amino acid metabolism may break the balance of muscle breakdown, protein synthesis, and overactivation of gluconeogenesis, which precedes the onset of T2DM.

**Aim of Study:**

This study was designed to identify the levels of nonessential amino acids in recently developed T2DM patients, which may help in the early diagnosis of the disease.

**Methods:**

The 180 individuals who participated in this study were categorized as healthy controls, prediabetes, and T2DM. Tests conducted for each participant included glucose using enzymatic methods, HbA1c and nonessential amino acids using ion‐exchange high‐performance liquid chromatography (HPLC); and insulin using a sandwich‐based electrochemiluminescence immunoassay (ECLIA). Insulin resistance was calculated using the HOMA‐IR formula.

**Results:**

Newly diagnosed T2DM patients had increased alanine, asparagine, aspartate, glutamate, and proline but decreased serine, arginine, cystine, glutamine, and glycine levels. Amino acid levels varied by obesity status, with alanine, aspartate, and serine rising with obesity, while asparagine, glutamate, arginine, and glycine decreased. Alanine, aspartate, and serine correlated positively with BMI, insulin, and HOMA‐IR, whereas asparagine, glutamate, and arginine showed negative correlations. Cystine and glycine correlated negatively with age. Strong positive and negative interrelationships existed among these amino acids.

**Conclusion:**

The study revealed that newly diagnosed T2DM and prediabetes are associated with increased levels of certain nonessential amino acids (alanine, asparagine, aspartate, glutamate, proline) and decreased serine, arginine, cystine, glutamine, and glycine compared to healthy controls. These amino acid levels were influenced by obesity and correlated with insulin resistance and BMI. Altered amino acid metabolism plays a role in early T2DM development and could serve as biomarkers for diagnosis and management.

## 1. Introduction

Type 2 diabetes mellitus (T2DM) is a complex metabolic disorder characterized by chronic hyperglycemia arising from insulin resistance and/or impaired insulin secretion [1]. Recent advances have highlighted the importance of amino acid metabolism in the pathogenesis and progression of T2DM [2]. Specifically, the dysregulation of plasma amino acids, including nonessential amino acids, has been increasingly recognized as an early metabolic signature associated with insulin resistance and glycemic control abnormalities in individuals with T2DM [3]. The dysregulation of plasma nonessential amino acids may reflect underlying metabolic imbalances involving gluconeogenesis, the urea cycle, and nitric oxide (NO) synthesis, which are critical for maintaining glucose homeostasis and vascular function [2]. Understanding these early metabolic changes could identify novel biomarkers for T2DM diagnosis and therapeutic targeting to improve glycemic control and prevent complications.

Nonessential amino acids (NEAAs) are five dispensable amino acids that are not necessary for most physiological states in a healthy adult because the human body is capable of making them, which include alanine (Ala), asparagine (Asn), aspartate (Asp), glutamate (Glu), and serine (Ser) [4].

Alanine induces gluconeogenesis in muscle as well as in liver and may be a potential biomarker for glucose synthesis in T2DM. It is clear that alanine can be generated in all organs, but larger amounts are generated in organs with a high energy turnover [5]. Glutamate concentrations are reported to be elevated in the liver and muscles in patients under T2DM conditions. In the liver, alanine and glutamine are increased in comparison with other tissues and organs [6].

Asparagine and aspartate can be converted to each other by corresponding enzymes with metabolism fluctuation [7]. Asparagine synthetase (ASNS) is expressed in many tissues, especially the pancreas, and catalyzes the conversion of aspartate to asparagine in an ATP‐dependent way, while asparaginase can catalyze the conversion of asparagine to aspartate. Asparagine and aspartate are found to be linked to metabolic disorder traits and metabolic pathways in humans [8].

L‐serine can be synthesized from 3‐phosphoglycerate and glycine. L‐serine is a substrate for glucose and protein synthesis, and it is a building block of phospholipids, particularly phosphatidylserine, and sphingolipids, such as ceramides, phosphosphingolipids, and glycosphingolipids, which are highly concentrated in all cell membranes [9]. D‐serine, synthesized in neurons by serine racemase from L‐serine, serves as a neuromodulator [10].

Arginine is a critical precursor for the synthesis of NO, a potent vasodilator meaning that alterations in circulating arginine concentrations are deeply tied to vascular endothelial health and pancreatic *β*‐cell secretion kinetics in diabetic phenotypes [2, 11]. Cystine, the oxidized dimer of cysteine, functions as a rate‐limiting contributor to cellular glutathione synthesis and antioxidant defense; its systematic depletion or imbalance has been strongly correlated with chronic systemic oxidative stress during the transition from prediabetes to manifest T2DM [2]. Conversely, tyrosine is an aromatic amino acid metabolized via separate hepatic pathways, which frequently demonstrates highly distinct regulatory dynamics under insulin‐resistant constraints compared to aliphatic or acidic NEAAs, making it an informative metabolic control marker in early diagnostic panels [12]. Furthermore, glutamine serves as a major interorgan nitrogen carrier and a fundamental substrate for renal gluconeogenesis, while glycine acts as a critical structural and protective anti‐inflammatory molecule whose circulating concentrations have consistently demonstrated strong inverse relationships with hepatic fat accumulation and peripheral insulin sensitivity [13, 14]. On the other hand, proline metabolism is directly interwoven with collagen turnover and tricarboxylic acid (TCA) cycle anaplerosis; its accumulation in plasma is often associated with compensatory mitochondrial adaptations under conditions of lipotoxicity and disrupted glucose clearance [12]. Consequently, expanding the metabolic profile to evaluate this collection of 11 NEAAs concurrently allows for a definitive assessment of whether early‐stage dysregulation is highly specific to distinct homeostatic pathways or reflects a generalized disruption of the broader circulating amino acid pool. The aim of this study is to investigate the dysregulation of plasma NEAAs as biomarkers in patients with early‐stage T2DM and to elucidate their potential role in the pathophysiology and metabolic disturbances associated with the disease.

## 2. Materials and Methods

### 2.1. Subjects and Sample Size

This study was conducted on a total of 180 human subjects, divided equally into three primary diagnostic groups based on their glycemic status (*n* = 60 volunteers per group): healthy controls, prediabetes, and newly diagnosed T2DM. To control for anthropometric confounders, a frequency matching (group matching) methodology was rigorously applied during participant selection. Participants were prospectively recruited and balanced such that each primary diagnostic arm contained an identical distribution of body weight strata. Specifically, within each group, an equal quota of 20 volunteers was strictly allocated into three BMI subcategories: normal weight (18.5–24.9 kg/m^2^), overweight (25.0–29.9 kg/m^2^), and obese (≥ 30 kg/m^2^). Age distribution was similarly frequency‐matched across the cohorts, resulting in structurally equivalent groups with no statistically significant differences in mean age (*p* = 0.921) or mean BMI (*p* = 0.473) as confirmed by one‐way ANOVA (Table 1).

**TABLE 1:** The anthropometric and biochemical parameters for the participants.

Variables	Control Mean ± SD (*n* = 60)	Prediabetes Mean ± SD (*n* = 60)	T2DM Mean ± SD (*n* = 60)	*F* value	*p* value
Age (years)	51.8 ± 13.55	52.75 ± 12.90	52.48 ± 11.68	0.09	0.921
BMI (kg/m^2^)	27.80 ± 4.13	28.77 ± 5.01	28.63 ± 4.89	0.75	0.473
Glucose (mg/dL)	85.5 ± 8.27	117.15 ± 4.27^a^	208.27 ± 73.70^c,e^	132.53	< 0.001
HbA1c (%)	5.01 ± 0.29	6.10 ± 0.22^a^	9.09 ± 1.60^c,e^	297.61	< 0.001
Insulin (μlU/mL)	7.79 ± 2.22	21.09 ± 5.09^a^	28.32 ± 6.85^c,e^	251.10	< 0.001
HOMA‐IR	1.64 ± 0.50	6.11 ± 1.53^a^	13.93 ± 4.41^c,e^	316.10	< 0.001

^a^Comparison between prediabetes and control groups was significant at *p* < 0.001.

^c^Comparison between T2DM and control groups was significant at *p* < 0.001.

^e^Comparison between T2DM and prediabetes groups was significant at *p* < 0.001.

The new diagnosis of prediabetes and T2DM were based on fasting serum glucose and HbA1c levels attended to the specialist advisory clinic at Basra City’s Al‐Fayhaa Teaching Hospital. The volunteers age ranged from 30 to 75 years. Information collected from each participant included name, age, sex, height, and weight. Blood samples were collected from all participants between April and August 2024. Venous blood (8 mL) was drawn in the morning after 9–12 h of fasting using a disposable butterfly needle and divided into three tubes.

First, 2 mL of blood was transferred into an EDTA K3–containing vacutainer tube for the estimation of HbA1c within 30 min. Second, 3 mL of blood was transferred into a gel‐ and clot activator–containing evacuated tube and stood for 30 min and then centrifuged for 15 min at 3500 rotations per minute (RPM) for the separation of serum for the estimation of glucose and insulin levels within 30 min. Third, 3 mL of blood was transferred into a lithium heparin‐containing vacutainer tube, then centrifuged at 3500 RPM for a period of 15 min for plasma separation, transferred into an Eppendorf tube, and then frozen at −80°C for the subsequent analysis of the nonamino acid profile. The frozen samples were thawed at room temperature (21°C) for 30 min until they were completely thawed and then centrifuged at 3500 RPM for 5 min before analysis.

The healthy control group consisted of 60 apparently healthy subjects balanced for sex, age, and BMI. Controls were recruited from family, friends, and hospital staff.

To confirm the statistical validity of this sample size, a *post hoc* power analysis was executed using G^∗^ Power software (Version 3.1.9.7) for a one‐way ANOVA across the three groups. Utilizing the observed means and standard deviations of the primary biomarkers (e.g., plasma alanine: 412.35 ± 40.19 μmol/L for controls, 622.30 ± 43.25 μmol/L for prediabetes, and 763.93 ± 67.14 μmol/L for T2DM), the calculated effect size (*f*) was exceptionally high. With a total sample size of 180 (*n* = 60 per group) and a significance level (*α*) of 0.05, the actual statistical power (1 − *β*) achieved was > 0.99, verifying that the study is optimally powered to avoid Type II errors and to ensure high reproducibility of the metabolic profiles.

### 2.2. Inclusion Criteria

Volunteers aged 30–75 years were included if they meet one of the following criteria: newly diagnosed T2DM (fasting serum glucose ≥ 126 mg/dL and HbA1c ≥ 6.5%), prediabetes (fasting serum glucose 110–125 mg/dL and HbA1c 5.7%–6.4%), or healthy controls (fasting serum glucose 70–< 110 mg/dL and HbA1c 4.5–< 5.7%).

### 2.3. Exclusion Criteria

Volunteers in the present study were excluded if they had kidney, pancreatic, cardiovascular, liver, or autoimmune diseases (including thyroid disorders and T1DM), were pregnant or lactating, or were taking medications (such as corticosteroids) known to influence glucose metabolism, no recent blood transfusions, surgeries, or biological agent intake.

### 2.4. Methods

Standing height and body weight were obtained using a stadiometer and a balance, respectively; this formula was utilized to determine the body mass index (BMI)—BMI = weight (kg) divided by height (m^2^).

Serum glucose has been determined by the enzymatic methods in a fully automated biochemistry analyzer (Spin 200 analyzer, Spinreact Company, Spain). Whole blood was tested for glycosylated hemoglobin A1c (HbA1c) by the ion‐exchange high‐performance liquid chromatography (HPLC) technique in a fully automated analyzer (D‐10 analyzer, Bio‐Rad Company, USA).

The sandwich‐based electrochemiluminescence immunoassay (ECLIA) technique was applied in a fully automated immunoassay instrument (Cobas e 411 analyzer, Roche business, Germany) to determine the serum insulin level. Insulin resistance was assessed using the homeostatic model assessment of the insulin resistance (HOMA‐IR) formula: HOMA‐IR is equivalent to fasting insulin (mIU/L) × fasting glucose (mg/dL)/405. Plasma amino acid concentrations were measured by the ion‐exchange HPLC technique applied in a fully automated system (Aracus Classic amino acid analyzer, membraPure Company, Germany)

### 2.5. Statistical Analysis

All data analysis was done using Version 26.0 of SPSS. The use of one‐way ANOVA allowed for the comparison of means between multiple groups, identifying statistically significant differences. Pearson’s correlation coefficients assessed the relationships between continuous variables. A significance threshold of *p* < 0.05 was used, meaning that any observed differences or associations with *p* values below this threshold are unlikely to be due to chance.

## 3. Results

Three equal‐number categories were formed from the 180 participants in the current study and matched in terms of anthropometric characteristics, such as age and BMI, with nonsignificant differences (*p* > 0.05). However, newly identified T2DM had statistically significant increases in glucose, HbA1c, insulin, and HOMA‐IR compared to prediabetes and healthy controls (*p* < 0.001), as presented in clinical baseline data in Table 1.

Table 2 shows the plasma levels of NEAAs across the study groups. Patients with newly diagnosed T2DM and prediabetes exhibited higher alanine, asparagine, aspartate, glutamate, and proline levels than healthy controls (*p* < 0.001); these levels were even greater in newly diagnosed T2DM than in prediabetes (*p* < 0.001). In contrast, serine, arginine, cystine, glutamine, and glycine levels decreased in patients with newly diagnosed T2DM and prediabetes compared to healthy controls (*p* < 0.001), as well as in newly diagnosed T2DM versus prediabetes (*p* < 0.001). Tyrosine levels showed no significant differences among the groups (*p* > 0.05).

**TABLE 2:** Plasma nonessential amino acid levels among study groups.

Nonessential AAs	Control Mean ± SD (*n* = 60)	Prediabetes Mean ± SD (*n* = 60)	T2DM Mean ± SD (*n* = 60)	*F* value	*p* value
Alanine (μmol/L)	412.35 ± 40.19	622.30 ± 43.25^a^	763.93 ± 67.14^c,e^	704.62	< 0.001
Asparagine (μmol/L)	48.80 ± 8.04	75.08 ± 9.10^a^	84.72 ± 8.51^c,e^	283.11	< 0.001
Aspartate (μmol/L)	3.97 ± 1.95	6.75 ± 1.63^a^	14.05 ± 3.64^c,e^	247.84	< 0.001
Glutamate (μmol/L)	78.35 ± 11.04	109.55 ± 10.98^a^	153.90 ± 20.18^c,e^	399.29	< 0.001
Serine (μmol/L)	89.95 ± 18.19	55.48 ± 8.72^a^	44.75 ± 8.51^c,e^	209.38	< 0.001
Arginine (μmol/L)	78.72 ± 17.94	59.48 ± 16.71^a^	27.55 ± 11.21^c,e^	165.44	< 0.001
Cystine (μmol/L)	65.20 ± 8.03	43.45 ± 9.33^a^	24.27 ± 9.16^c,e^	320.69	< 0.001
Tyrosine (μmol/L)	60.50 ± 11.12	59.85 ± 10.33	61.25 ± 11.13	0.25	0.780
Glutamine (μmol/L)	78.35 ± 12.48	57.55 ± 9.44^a^	43.35 ± 7.86^c,e^	181.90	< 0.001
Glycine (μmol/L)	71.77 ± 10.01	52.80 ± 8.92^a^	33.17 ± 9.10^c,e^	255.46	< 0.001
Proline (μmol/L)	113.27 ± 20.88	131.25 ± 26.17^a^	143.42 ± 26.53^c,f^	22.70	< 0.001

^a^Comparison between prediabetes and control groups was significant at *p* < 0.001.

^c^Comparison between T2DM and control groups was significant at *p* < 0.001.

^e^Comparison between T2DM and prediabetes groups was significant at *p* < 0.001.

^f^Comparison between T2DM and prediabetes groups was significant at *p* < 0.05 as determined by one‐way ANOVA Tukey HSD.

Based on the results of plasma NEAA levels in T2DM based on the BMI in Table 3, circulating levels of alanine, aspartate, and serine were elevated in obese patients with newly diagnosed T2DM compared to those who were overweight or normal weight (*p* < 0.001). Levels of these amino acids were also higher in the overweight group than in the normal‐weight group (*p* < 0.001). In contrast, asparagine and arginine levels were lower in the obese group than in the overweight and normal‐weight groups (*p* < 0.001), whereas asparagine did not differ significantly between the overweight and normal‐weight groups (*p* > 0.05).

**TABLE 3:** Plasma nonessential amino acid levels in T2DM based on the body mass index.

Nonessential AAs	Normal weight Mean ± SD (*n* = 20)	Overweight Mean ± SD (*n* = 20)	Obese Mean ± SD (*n* = 20)	*F* value	*p* value
Alanine (μmol/L)	690.25 ± 24.80	758.20 ± 23.10^a^	843.35 ± 21.46^c,e^	219.36	< 0.001
Asparagine (μmol/L)	94.55 ± 2.89	84.70 ± 2.49	74.90 ± 2.61^c,e^	270.41	< 0.001
Aspartate (μmol/L)	10.00 ± 1.45	13.95 ± 1.47	18.20 ± 1.20^c,e^	177.27	< 0.001
Glutamate (μmol/L)	162.10 ± 19.79	159.75 ± 16.72	139.85 ± 16.71^d,f^	9.44	< 0.001
Serine (μmol/L)	35.05 ± 2.63	45.00 ± 2.73^a^	54.20 ± 4.16^c,e^	173.66	< 0.001
Arginine (μmol/L)	39.10 ± 6.13	28.65 ± 5.79^a^	14.90 ± 3.04^c,e^	110.04	< 0.001
Cystine (μmol/L)	24.10 ± 8.48	20.80 ± 8.77	27.90 ± 9.23^f^	3.24	0.047
Tyrosine (μmol/L)	61.35 ± 12.97	58.30 ± 10.34	64.10 ± 9.58	1.38	0.261
Glutamine (μmol/L)	45.30 ± 8.93	41.05 ± 7.72	43.70 ± 6.55	1.52	0.228
Glycine (μmol/L)	38.45 ± 7.50	29.45 ± 8.99^b^	31.60 ± 8.57^d^	6.29	0.003
Proline (μmol/L)	146.80 ± 28.28	139.10 ± 27.16	144.35 ± 24.85	0.43	0.652

^a^Comparison between overweight and normal‐weight groups was significant at *p* < 0.001.

^b^Comparison between overweight and normal‐weight groups was significant at *p* < 0.05.

^c^Comparison between obese and normal‐weight groups was significant at *p* < 0.001.

^d^Comparison between obese and normal‐weight groups was significant at *p* < 0.05.

^e^Comparison between obese and overweight groups was significant at *p* < 0.001.

^f^Comparison between obese and overweight groups was significant at *p* < 0.05 as determined by one‐way ANOVA Tukey HSD.

Plasma glutamate was reduced in the obese group relative to the overweight and normal‐weight groups (*p* < 0.05), but showed no significant difference between the overweight and normal‐weight groups (*p* > 0.05). Cystine levels were higher in the obese group than in the overweight group (*p* < 0.05); however, cystine did not differ significantly between the overweight or obese groups and the normal‐weight group (*p* > 0.05). Glycine levels, conversely, were lower in both the obese and overweight groups compared to the normal‐weight group (*p* < 0.05), with no significant difference between the obese and overweight groups (*p* > 0.05). Finally, tyrosine, glutamine, and proline levels showed no significant differences across BMI groups (*p* > 0.05).

The correlation analysis in Table 4, examining NEAAs and key clinical parameters in newly diagnosed T2DM patients, revealed the following patterns. Age showed strong inverse associations with cystine and proline (*p* < 0.001); all other relationships were nonsignificant (*p* > 0.05). BMI exhibited strong positive correlations with alanine, aspartate, and serine (*p* < 0.001), alongside strong inverse correlations with asparagine and arginine (*p* < 0.001) and weak inverse correlation with glutamate (*p* < 0.001); remaining associations were nonsignificant (*p* > 0.05). Neither glucose nor HbA1c levels had significant links with any NEAAs (*p* > 0.05). Insulin displayed moderate positive correlations with alanine and aspartate (*p* < 0.001), weak positive correlation with serine (*p* < 0.001), moderate inverse correlation with asparagine (*p* < 0.001), and inverse correlation with arginine (*p* < 0.001); other associations were nonsignificant (*p* > 0.05). HOMA‐IR demonstrated a moderate positive correlation with alanine (*p* < 0.001), weak positive correlations with aspartate and serine, a weak negative correlation with arginine (*p* < 0.001), asparagine, and glutamate (*p* < 0.05); all others were nonsignificant (*p* > 0.05).

**TABLE 4:** Correlation between nonessential amino acids and basic parameters in T2DM.

Parameters	Correlation	Alanine	Asparagine	Aspartate	Glutamate	Serine	Arginine	Cystine	Tyrosine	Glutamine	Glycine	Proline
Age	*r*	−0.098	0.075	−0.038	−0.009	0.038	−0.013	−0.891	−0.183	−0.160	−0.850	−0.046
	*p* value	0.459	0.567	0.775	0.948	0.776	0.923	0.000	0.161	0.222	0.000	0.725

BMI	*r*	0.844	−0.871	0.839	−0.450	0.863	−0.803	0.193	0.156	−0.162	−0.205	−0.067
	*p* value	0.000	0.000	0.000	0.000	0.000	0.000	0.139	0.233	0.216	0.116	0.611

HbA1c	*r*	0.001	0.011	−0.072	−0.081	−0.120	−0.025	0.064	−0.100	−0.170	0.052	0.094
	*p* value	0.996	0.934	0.583	0.540	0.363	0.849	0.627	0.447	0.193	0.693	0.477

Glucose	*r*	0.056	0.059	−0.020	−0.285	−0.055	−0.035	−0.031	0.062	−0.008	0.065	0.102
	*p* value	0.670	0.655	0.882	0.027	0.676	0.790	0.817	0.637	0.951	0.624	0.437

Insulin	*r*	0.530	−0.507	0.501	−0.085	0.445	−0.477	0.142	0.031	−0.028	−0.131	−0.119
	*p* value	0.000	0.000	0.000	0.519	0.000	0.000	0.279	0.814	0.831	0.317	0.364

HOMA‐IR	*r*	0.532	−0.384	0.400	−0.326	0.360	−0.457	0.014	0.054	−0.021	−0.153	−0.012
	*p* value	0.000	0.002	0.002	0.011	0.005	0.000	0.917	0.683	0.872	0.243	0.929

Table 5 depicts the correlations among circulating NEAAs in patients newly diagnosed with T2DM. Alanine exhibited strong positive associations with aspartate and serine (*p* < 0.001), as well as strong inverse associations with asparagine and glutamate (*p* < 0.001); it also showed a weak inverse link with serine (*p* < 0.05), while all other associations were nonsignificant (*p* > 0.05).

**TABLE 5:** Correlation analysis between nonessential amino acids in T2DM.

Nonessential AAs	Correlation	Alanine	Asparagine	Aspartate	Glutamate	Serine	Arginine	Cystine	Tyrosine	Glutamine	Glycine	Proline
Alanine	*r*	1	−0.898	0.896	−0.415	0.883	−0.833	0.180	0.070	−0.057	−0.249	−0.119
	*p* value		0.000	0.000	0.001	0.000	0.000	0.169	0.594	0.668	0.055	0.364

Asparagine	*r*	−0.898	1	−0.910	0.423	−0.880	0.821	−0.200	−0.025	0.057	0.261	0.012
	*p* value	0.000		0.000	0.001	0.000	0.000	0.125	0.851	0.665	0.044	0.927

Aspartate	*r*	0.896	−0.910	1	−0.403	0.881	−0.810	0.140	0.068	−0.095	−0.298	−0.137
	*p* value	0.000	0.000		0.001	0.000	0.000	0.284	0.604	0.468	0.021	0.296

Glutamate	*r*	−0.415	0.423	−0.403	1	−0.429	0.493	−0.020	−0.009	0.107	0.112	0.024
	*p* value	0.001	0.001	0.001		0.001	0.000	0.879	0.947	0.417	0.393	0.856

Serine	*r*	0.883	−0.880	0.881	−0.429	1	−0.802	0.043	−0.029	−0.035	−0.387	−0.054
	*p* value	0.000	0.000	0.000	0.001		0.000	0.746	0.827	0.793	0.002	0.685

Arginine	*r*	−0.833	0.821	−0.810	0.493	−0.802	1	−0.148	−0.200	0.119	0.291	0.052
	*p* value	0.000	0.000	0.000	0.000	0.000		0.258	0.125	0.364	0.024	0.692

Cystine	*r*	0.180	−0.200	0.140	−0.020	0.043	−0.148	1	0.279	0.114	0.805	0.107
	*p* value	0.169	0.125	0.284	0.879	0.746	0.258		0.031	0.388	0.000	0.415

Tyrosine	*r*	0.070	−0.025	0.068	−0.009	−0.029	−0.200	0.279	1	−0.090	0.238	0.022
	*p* value	0.594	0.851	0.604	0.947	0.827	0.125	0.031		0.495	0.067	0.866

Glutamine	*r*	−0.057	0.057	−0.095	0.107	−0.035	0.119	0.114	−0.090	1	0.155	0.221
	*p* value	0.668	0.665	0.468	0.417	0.793	0.364	0.388	0.495		0.237	0.090

Glycine	*r*	−0.249	0.261	−0.298	0.112	−0.387	0.291	0.805	0.238	0.155	1	0.121
	*p* value	0.055	0.044	0.021	0.393	0.002	0.024	0.000	0.067	0.237		0.356

Proline	*r*	−0.119	0.012	−0.137	0.024	−0.054	0.052	0.107	0.022	0.221	0.121	1
	*p* value	0.364	0.927	0.296	0.856	0.685	0.692	0.415	0.866	0.090	0.356	

Asparagine displayed strong inverse associations with aspartate and serine (*p* < 0.001), alongside a strong positive association with arginine (*p* < 0.001) and a weak positive link with glutamate (*p* < 0.05); remaining associations were nonsignificant (*p* > 0.05).

Aspartate revealed a strong positive association with serine (*p* < 0.001), a strong inverse association with arginine (*p* < 0.001), and a weak inverse link with glutamate (*p* < 0.05); all other associations were nonsignificant (*p* > 0.05).

Glutamate showed a weak negative association with serine (*p* < 0.05) and a weak positive association with arginine (*p* < 0.05); other associations were nonsignificant (*p* > 0.05).

Serine exhibited a strong negative association with arginine (*p* < 0.001) and a weak negative link with glycine (*p* < 0.05); remaining associations were nonsignificant (*p* > 0.05).

Arginine demonstrated a weak positive association with glycine (*p* < 0.05); all other associations were nonsignificant (*p* > 0.05).

Cystine showed a weak positive association with tyrosine (*p* < 0.05) and a strong positive association with glycine (*p* < 0.001); other associations were nonsignificant (*p* > 0.05).

Tyrosine, glutamine, and glycine exhibited nonsignificant associations with one another (*p* > 0.05).

## 4. Discussion

Matching the groups on anthropometric characteristics such as age and BMI with no significant differences (*p* > 0.05) strengthens the conclusions by minimizing confounding effects from these known metabolic predictors. In this study, the implementation of a frequency matching design across a stratified 3 × 3 grid ensured that the baseline distribution of adiposity and age was statistically uniform among the healthy control, prediabetes, and diabetic cohorts. This strict group‐level balancing allows for a clearer and more robust interpretation, demonstrating that the observed dysregulation in plasma NEAA profiles is primarily a reflection of the patients’ underlying glycemic and insulin resistance status, rather than being driven by disparities in age or BMI distribution between the studied groups.

Elevated fasting glucose and HbA1c levels in newly diagnosed T2DM are well‐documented indicators of poor glycemic control and chronic hyperglycemia, which underly the pathophysiology of T2DM [15, 16]. HbA1c, in particular, reflects average blood glucose levels over the preceding 2‐3 months, providing a robust clinical benchmark for diagnosing and monitoring diabetes progression, and its significant elevation alongside fasting glucose in the diabetic group affirms the chronic nature of the hyperglycemic state in these patients [17, 18]. Insulin levels and HOMA‐IR are crucial markers illustrating the extent of insulin resistance and pancreatic β‐cell compensatory response [19]. In early T2DM, it is common to observe increased insulin levels as pancreatic β‐cells augment secretion to overcome peripheral insulin resistance [20]. HOMA‐IR, calculated from fasting glucose and insulin, quantitatively estimates insulin resistance, which is a central mechanism in the pathogenesis of T2DM [21]. The significant elevation of HOMA‐IR in the newly diagnosed group denotes marked insulin resistance compared to prediabetes and nondiabetic controls, corroborating its role in disease onset, and this insulin resistance leads to the characteristic metabolic impairments of glucose uptake and utilization seen in T2DM patients [22, 23].

Several studies have demonstrated that altered plasma amino acid profiles are closely associated with insulin resistance and the onset of T2DM [13, 24]. Elevated levels of alanine, asparagine, aspartate, and glutamate in diabetes likely reflect enhanced gluconeogenesis and disrupted intermediary metabolism, as these NEAAs serve as major substrates for glucose production in the liver [25]. Amino acids such as alanine and glutamate are closely linked to the anaplerotic and cataplerotic pathways of the TCA cycle, which are dysregulated in T2DM, contributing to increased hepatic glucose output and hyperglycemia [13, 25]. In contrast, the decrease in serine observed in T2DM and prediabetes patients is consistent with prior work highlighting reduced serine levels as a potential biomarker of metabolic dysfunction. Serine plays important roles in one‐carbon metabolism, antioxidant defense, and lipid metabolism, and lower serine availability may impair cellular redox balance and insulin signaling pathways [25]. Decreased serine has also been positively correlated with insulin resistance and may reflect altered protein and amino acid metabolism in diabetes progression [13]. Moreover, the significant differences between T2DM and prediabetes in these amino acid levels underscore the progressive metabolic deterioration as insulin resistance worsens and β‐cell dysfunction ensues during the transition to overt diabetes [24]. The observation that patients with newly diagnosed T2DM and prediabetes exhibit lower plasma levels of arginine, cysteine, glutamine, and glycine compared to healthy controls and that these levels were even lower in newly developed T2DM compared to prediabetes. L‐arginine serves as the sole substrate for NO synthesis via nitric oxide synthase (NOS). NO is a crucial vasodilator that modulates endothelial function and insulin‐mediated glucose uptake in skeletal muscle, and deficiency in arginine can exacerbate inflammation and IR, contributing to T2DM development [26]. Although less extensively studied, cysteine levels have been reported to be altered in diabetic states. Pilot study detects cysteine in controls and diabetic subjects, suggesting its involvement in amino acid metabolism changes in diabetes [27]. However, detailed mechanistic insights remain limited. The progressive decline in plasma glutamine levels reflects accelerated utilization by the liver and kidneys for gluconeogenesis to compensate for perceived intracellular glucose deprivation caused by insulin resistance. Additionally, glutamine stimulates glucagon‐like peptide‐1 (GLP‐1) secretion from intestinal L‐cells; its depletion, therefore, compromises incretin‐mediated insulin secretion, worsening postprandial glycemic control [28, 29]. The reduction of circulating glycine in early T2DM is primarily attributed to its overconsumption in response to heightened oxidative stress (for GSH production) and its accelerated clearance via upregulated hepatic gluconeogenic pathways. Lower glycine levels remove a protective buffer, directly promoting systemic inflammation and worsening tissue insulin resistance [30]. However, proline levels were higher in newly diagnosed T2DM compared to healthy subjects and prediabetes, and the levels of proline were elevated in prediabetes more than in healthy subjects. Elevated proline levels are indicative of tissue remodeling, collagen degradation, or a shift in mitochondrial energetics under conditions of metabolic stress. Proline accumulation contributes to an altered intracellular redox state NAD^+^/NADH and NADP^+^/NADPH ratios, generating reactive oxygen species (ROS) that contribute to mitochondrial dysfunction and downstream insulin resistance in metabolic tissues like the liver and skeletal muscle [31]. These changes in amino acid profiles may not only serve as indicators for early detection but also provide mechanistic insights into disease pathogenesis, offering potential targets for intervention and personalized treatment strategies [13, 25].

Circulating levels of alanine, aspartate, and serine were significantly higher in the obese group compared to both overweight and normal‐weight groups, with the overweight group also showing elevated levels relative to normal‐weight patients (*p* < 0.001). This pattern is consistent with metabolic alterations associated with obesity and T2DM. Elevated alanine and aspartate levels have been reported in obese individuals and those with T2DM, reflecting altered amino acid metabolism and increased gluconeogenic substrate availability in these conditions [32, 33]. Alanine, produced through muscle protein breakdown and transamination, is a key glucogenic amino acid contributing to hepatic glucose production, which is often upregulated in obesity and insulin resistance states [32, 34]. Similarly, aspartate’s elevation may be related to disruptions in the TCA cycle and nitrogen metabolism, which are altered in obesity and T2DM [33]. Conversely, asparagine levels were significantly lower in the obese group compared to overweight and normal‐weight groups, with no significant difference between overweight and normal‐weight patients. The reduction of asparagine in obesity has been noted in some studies and may reflect enhanced catabolism or altered amino acid transporter activity related to metabolic dysfunction [35]. Asparagine plays a role in nitrogen balance and cellular metabolism, and its reduction could indicate metabolic stress or dysregulation in obese T2DM patients [35]. Plasma glutamate was similarly lower in the obese group compared to overweight and normal‐weight patients, although glutamate levels did not differ significantly between overweight and normal‐weight groups. Glutamate is involved in multiple metabolic pathways including amino acid transamination, and its decrease could be linked to altered nitrogen and energy metabolism in obesity and T2DM [32, 35]. The lowered glutamate levels in obesity might be associated with impaired mitochondrial function, as glutamate is crucial for mitochondrial energy metabolism. Circulating levels of arginine were lower in the obese compared to the overweight and normal weight, and these levels were also decreased in the overweight compared to the normal weight of the patients with recently identified T2DM. Metabolomic analyses in T2DM patients show downregulated arginine levels in obese diabetic groups compared to obese nondiabetic controls, suggesting that T2DM exacerbates arginine depletion beyond the effect of obesity alone [36]. Elevated arginase activity has been linked to obesity‐related hypertension and IR, conditions frequently accompanying T2DM [37]. This enzymatic activity could contribute to the significant decrease in arginine with increasing BMI categories. Rather than baseline circulating arginine levels, arginine supplementation has been shown to improve insulin sensitivity and reduce adiposity in obese and diabetic models [38, 39]. This complexity suggests that arginine levels are part of a multifactorial metabolic disturbance in obesity and T2DM.

The correlation analysis between NEAAs and clinical parameters in newly diagnosed T2DM patients reveals distinct patterns that align with metabolic dysfunction characteristic of the disease. Alanine, aspartate, and serine showed no significant associations with age, HbA1c, or glucose levels, indicating that these amino acids may be less influenced by glycemic status or aging in early T2DM. However, alanine and aspartate demonstrated strong positive correlations with BMI and insulin levels, reflecting their potential involvement in obesity‐related insulin resistance mechanisms [40, 41]. Their significant positive associations with HOMA‐IR further support a role in insulin resistance modulation [42]. In contrast, asparagine and glutamate exhibited significant negative correlations with BMI, insulin, HOMA‐IR, and glucose, implying a divergent metabolic role potentially related to protective or compensatory pathways in glucose homeostasis and adiposity [43, 44]. The negative relationship between these amino acids and insulin resistance markers aligns with previous findings suggesting that disrupted amino acid metabolism is intricately linked to metabolic health and diabetes progression [45]. The absence of associations with age and HbA1c indicates that these relationships may primarily reflect current metabolic status rather than chronic glycemic exposure or aging. These results underscore the complexity of amino acid metabolic perturbations in early T2DM, where specific NEAAs differentially correlate with markers of obesity, insulin secretion, and insulin sensitivity. Alanine and aspartate, linked to gluconeogenesis and nitrogen metabolism, may contribute to or reflect enhanced metabolic stress in obese, insulin‐resistant states [46]. By contrast, asparagine and glutamate’s negative associations may indicate their depletion or altered utilization in metabolic dysregulation, consistent with studies highlighting glutamate pathways in insulin signaling and mitochondrial function [47].

Serine shows an exceptionally strong positive correlation with BMI. Because higher adiposity (BMI) naturally drives greater insulin resistance, fasting hyperinsulinemia, and elevated HOMA‐IR, the positive correlations between serine and insulin/HOMA‐IR are heavily mediated by this obesity‐dependent subgroup progression within the diabetic cohort. L‐serine serves as a vital substrate for protein synthesis and the generation of phospholipids and sphingolipids (such as ceramides) critical for cell membrane integrity [5]. In obese diabetic individuals, the combination of advanced insulin resistance and lipid overload (lipotoxicity) triggers a well‐documented compensatory upregulation of endogenous serine synthesis paths (such as the synthesis from 3‐phosphoglycerate and glycine) to meet the heightened demands of membrane remodeling and cellular defense [5]. Arginine was significantly negatively associated with BMI, insulin, and HOMA‐IR. L‐arginine improves insulin sensitivity by enhancing NO‐mediated vasodilation, which increases skeletal muscle glucose uptake [48]. This mechanism reduces compensatory hyperinsulinemia and lowers HOMA‐IR scores [49]. Concurrently, arginine promotes mitochondrial biogenesis and fatty acid oxidation in adipose tissue via the upregulation of peroxisome proliferator–activated receptor‐gamma coactivator‐1 alpha (PGC‐1α), leading to reduced visceral adiposity and BMI [50]. One study demonstrated that arginine supplementation decreases WC by 2.7 cm compared to diet/exercise alone (*p* < 0.0001) [51]. The negative relationship between cystine and age in newly diagnosed T2DM patients could indicate that older patients have a diminished capacity to maintain cystine/cysteine balance, possibly due to impaired renal function or altered amino acid metabolism [52]. Glycine levels have been found to be significantly negatively associated with age. Glycine is a precursor for GSH, a critical antioxidant that counters oxidative stress caused by hyperglycemia‐induced ROS. Insufficient glycine can limit GSH synthesis, exacerbating oxidative damage in diabetic tissues [13, 53]. This decline is possibly due to reduced glycine synthesis or increased utilization in aging‐related oxidative stress and inflammation pathways [54].

Alanine, asparagine, and glutamate are interconnected through transamination reactions catalyzed by aminotransferases, which are crucial in amino acid and glucose metabolism; ALT catalyzes the reversible transamination between alanine and 2‐oxoglutarate to form pyruvate and glutamate. Similarly, AST catalyzes the reversible transamination between aspartate and 2‐oxoglutarate to form oxaloacetate and glutamate. These reactions link the metabolism of alanine, asparagine (derived from aspartate), and glutamate with gluconeogenesis and nitrogen metabolism, especially in the liver [55]. Alanine is a key gluconeogenic amino acid, serving as a substrate for hepatic glucose production. Its increased formation from glutamate and pyruvate in T2DM can exacerbate hyperglycemia. Conversely, asparagine’s reduction may indicate impaired amino acid homeostasis, which has been linked to IR and metabolic syndrome [56]. Glutamate plays a central role as an amino group donor and acceptor in these transamination reactions, influencing the levels of alanine and asparagine. Elevated glutamate can increase the transamination of pyruvate to alanine, promoting gluconeogenesis, a process often upregulated in T2DM [24]. Glutamate may drive alanine production via transamination, while asparagine levels decrease due to altered amino acid metabolism or increased utilization in gluconeogenesis or other pathways [57]. The positive correlation between alanine and aspartate may be explained by their roles in amino acid metabolism and transamination reactions, where ALT catalyzes the reversible conversion between alanine and pyruvate, and AST catalyzes similar reactions involving aspartate and oxaloacetate, and alterations in these enzymes′ activities and amino acid levels reflect metabolic disturbances in T2DM [58, 59]. Serine’s positive correlation with alanine may relate to its involvement in gluconeogenesis and one‐carbon metabolism, processes often disrupted in diabetes. Reduced serine levels have been linked to the development of metabolic syndrome and diabetes progression [2]. The interplay among these amino acids reflects broader metabolic dysregulation in newly diagnosed T2DM, highlighting potential biomarkers for disease progression and targets for therapeutic intervention.

Asparagine is a glucogenic amino acid that is metabolically interconvertible with aspartate through enzymatic reactions, notably catalyzed by ASNS, which synthesizes asparagine from aspartate, ATP, and ammonia [60]. This metabolic interchange is critical in cellular nutrition and energy metabolism, particularly under glucose deficiency and amino acid disturbances common in T2DM [13]. Prospective observational study identifies plasma asparagine as a protective biomarker against diabetes, negatively correlating with fasting insulin, while aspartate is inversely associated with fasting glucose [13]. However, another study suggests that an elevated asparagine‐to‐aspartate ratio increases T2DM risk, especially in women aged over 50 years [57, 61]. This discrepancy may be explained by disease stage: Early diabetes or prediabetes may feature asparagine deficiency with compensatory ASNS upregulation leading to excess asparagine and aspartate deficiency later, contributing to hyperglycemia onset [13]. Asparagine can inhibit AMPK phosphorylation and activate mTORC1 signaling, promoting IR and beta‐cell dysfunction, pathways central to T2DM development [13]. Serine metabolism disturbances contribute to T2DM pathogenesis by impairing insulin secretion and sensitivity, and reduced serine availability leads to the accumulation of cytotoxic deoxysphingolipids, which damage pancreatic beta cells and disrupt glucose homeostasis [13]. This likely reflects metabolic shifts where increased conversion of asparagine to aspartate lowers circulating asparagine levels, while serine depletion occurs concurrently due to impaired synthesis and increased utilization under diabetic metabolic stress [13]. A large population study found glutamate to be the metabolite most strongly associated with incident T2DM, whereas asparagine was inversely associated with T2DM and coronary artery disease (CAD) risk; this suggests that glutamate elevation and asparagine depletion may be early metabolic markers preceding clinical T2DM [62]. The metabolic pathways involving asparagine and glutamate are interconnected within amino acid metabolism and the urea cycle. Specifically, N‐acetyl asparagine, a derivative of asparagine, has been linked to cardiovascular risk markers in diabetic patients, highlighting the involvement of asparagine‐related metabolism in T2DM complications [63]. Some studies have found that higher levels of glutamate are linked to changes in asparagine metabolism in T2DM and its complication, suggesting that they are connected [63].

Conversely, aspartate levels do not always parallel glutamate; some analyses report no significant difference in aspartate levels between T2DM patients and controls, suggesting a complex regulatory mechanism [64]. The negative correlation between aspartate and glutamate in newly diagnosed T2DM patients may indicate a metabolic shift where increased glutamate is accompanied by decreased aspartate availability or altered transamination dynamics. This could reflect impaired amino acid metabolism linked to IR and beta‐cell dysfunction, as glutamate is known to regulate insulin secretion negatively when elevated. The strong positive correlation between aspartate and serine suggests coordinated regulation or shared metabolic pathways that become dysregulated in T2DM; both amino acids may be upregulated as part of a compensatory response to metabolic stress or altered gluconeogenesis and amino acid catabolism in diabetes. This relationship may also reflect their roles in nitrogen balance and intermediary metabolism, which are often disrupted in diabetic patients [64]. A study assessing plasma amino acids in T2DM patients found that serine concentrations were significantly decreased, whereas glutamate concentrations were significantly increased compared to healthy controls [2]. The negative correlation may reflect disrupted amino acid metabolism linked to IR and glucose dysregulation in newly diagnosed T2DM patients [24]. The negative correlation between glutamate and serine, along with their respective associations with diabetic markers, has been observed in diverse populations, including Japanese and Middle Eastern cohorts, supporting the robustness of this metabolic pattern in T2DM [24].

Despite the significant findings regarding plasma NEAA dysregulation in early‐stage T2DM, several limitations must be explicitly acknowledged. First, this study utilized a cross‐sectional design, which inherently limits our ability to establish temporal or causal relationships between systemic amino acid alterations and the pathogenesis of insulin resistance or early‐stage diabetes. Prospective longitudinal cohorts are required to confirm whether these metabolic changes precede or result from early glycemic deterioration. Second, although our sample size of 180 participants was strictly balanced across a stratified grid (3 × 3 design) and demonstrated exceptional statistical power (> 99%) in post hoc testing for primary biomarkers, it remains a single‐center cohort. Larger, multicenter international investigations would enhance the external validity of these metabolic signatures. Third, the potential for residual confounding cannot be entirely omitted. While we rigorously applied an explicit list of exclusion criteria and frequency‐matched the groups for key anthropometric characteristics (age and BMI) to eliminate major confounding, other unmeasured lifestyle factors such as precise daily dietary macronutrient composition, physical activity indices, and hidden genetic variations could still subtly influence systemic amino acid concentrations. Finally, regarding generalizability, because all volunteers were prospectively recruited from a specialized clinical setting in Basra Governorate, Iraq, the metabolic profiles observed primarily reflect the baseline characteristics of this specific regional population. Extrapolating these findings to other ethnically, socioeconomically, or geographically distinct populations should be approached with caution until validated by broader global metabolic studies.

## 5. Conclusion

The study found that people with newly diagnosed T2DM and prediabetes have higher levels of certain NEAAs (alanine, asparagine, aspartate, glutamate, proline) and lower levels of serine, arginine, cystine, glutamine, and glycine compared to healthy individuals.

Alanine, aspartate, and serine exhibited a profound stepwise increase across BMI strata (normal weight < overweight < obese) and correlated strongly and positively with BMI, fasting insulin, and HOMA‐IR scores. Conversely, asparagine, glutamate, and arginine were significantly lower in obese phenotypes and demonstrated negative correlations with indices of adiposity and insulin resistance, underscoring their progressive depletion or accelerated consumption during metabolic stress. Cystine and glycine levels demonstrated strict inverse associations with age, suggesting a diminished capacity to counter hyperglycemia‐induced oxidative stress in older patient demographics. These findings highlight the potential of amino acid profiles as biomarkers for early T2DM and suggest that obesity influences amino acid metabolism in T2DM.

## Funding

No funding was received for this manuscript.

## Ethics Statement

This study received ethical approval from the Medical Ethics Committee of the Middle Technical University (MEC No.: 075 on January 13, 2024) at Baghdad Governorate. In addition, this study was reviewed and approved by the Research Committee of Basra Health Directorate (Approval No.: 1094 on 18 March, 2024) at Basra Governorate. The subjects provided written consent to participate in this investigation. The Helsinki Declaration was fully followed in the conduct of this study.

## Consent

Please see the Ethics Statement.

## Conflicts of Interest

The authors declare no conflicts of interest.

## Data Availability

The datasets generated and/or analyzed during the current study are available from the corresponding author upon reasonable request. Public access is restricted due to ethical and privacy considerations.
